# Ileocecocolic B-Cell Lymphoma Mimicking an Intestinal Neuroendocrine Tumor in a Cat: A Diagnostic Pitfall

**DOI:** 10.3390/vetsci13080731

**Published:** 2026-07-24

**Authors:** Ha-Neul Cho, Hyo-Sung Kim, Ki-Jung Kim, Hwi-Yool Kim

**Affiliations:** 1 Department of Veterinary Surgery, College of Veterinary Medicine, Konkuk University, 120 Neungdong-ro, Seoul 05029, Republic of Korea; 2 Department of Veterinary Clinical Pathology, College of Veterinary Medicine, Konkuk University, 120 Neungdong-ro, Seoul 05029, Republic of Korea; 3 The Joeun Animal Medical Center, Seoul 08631, Republic of Korea

**Keywords:** cat, B-cell lymphoma, neuroendocrine tumor-like morphology, immunophenotyping, ileocecocolic junction, full-thickness biopsy

## Abstract

An 8-year-old, 3.3 kg spayed female Korean Shorthair cat presented with chronic vomiting, waxing and waning hyporexia, and weight loss. Ultrasonography revealed a 1.5 × 1.6 cm ileocecocolic junction (ICCJ) mass and multiple colonic nodules. Fine-needle aspiration biopsy yielded predominantly oval cells with loosely cohesive clustering, and an intestinal neuroendocrine tumor (NET) was favored. Segmental resection of the distal ileum, ICCJ, and proximal colon with stapled side-to-side ileocolic anastomosis was performed. Histopathology also favored NET because the lesion consisted of relatively monomorphic round-to-polygonal cells with eosinophilic to chromophobic granular cytoplasm. Neoplastic cells showed strong CD20 immunoreactivity and nuclear PAX5 labeling but lacked CD3 immunoreactivity, with CD3-positive lymphocytes mainly peripheral to the neoplastic population. Labeling for chromogranin A, synaptophysin, and NSE was absent. These findings led to reclassification as B-cell lymphoma with NET-like morphology, most consistent with an intermediate-cell type. Postoperatively, vomiting improved and resolved after cyclophosphamide, doxorubicin, vincristine, and prednisolone-based chemotherapy was initiated. At approximately 6 months after surgery, body weight remained stable, no postoperative complications were identified, and no discrete recurrent mass was detected. This case demonstrates that feline enteric B-cell lymphoma may rarely exhibit NET-like morphology and be misclassified on cytology and routine histopathology without immunophenotypic confirmation.

## Simple Summary

Chronic vomiting in cats has many possible causes. When diagnostic imaging reveals an intestinal wall mass, neoplasia becomes an important concern. In this case, a cat with long-standing vomiting had a mass at the ileocecocolic junction. Cytology and routine histopathology both suggested a neuroendocrine tumor, which is rare in cats. Subsequent immunohistochemistry, however, showed that the lesion was a B-cell lymphoma. The cat underwent surgical resection of the dominant mass followed by systemic chemotherapy, and vomiting resolved after treatment. This case shows that feline intestinal B-cell lymphoma can mimic a neuroendocrine tumor on cytology and routine histology. Therefore, when a feline intestinal mass has a neuroendocrine tumor appearance, full-thickness tissue sampling and immunohistochemical confirmation should be strongly considered to ensure accurate diagnosis and appropriate treatment planning.

## 1. Introduction

Chronic vomiting is a common clinical presentation in cats; however, when diagnostic imaging identifies an intestinal wall mass, neoplasia becomes an important differential diagnosis. Feline lymphoma remains one of the most clinically important neoplastic diseases in cats, and, with the relative decline of feline leukemia virus (FeLV)-associated lymphoma, alimentary lymphoma has become an increasingly prominent presentation [1]. In clinical practice, feline alimentary lymphoma is commonly conceptualized as low-grade alimentary lymphoma, intermediate- to high-grade alimentary lymphoma, and the less common large granular lymphocyte lymphoma [2,3]. Within this spectrum, low-grade small-intestinal lymphoma is usually characterized by a mucosal small-cell T-cell phenotype, whereas transmural or focal mass-forming disease is more typical of intermediate- to high-grade alimentary lymphoma, which can be of either T- or B-cell lineage [2,3,4]. Although relatively uncommon, feline gastrointestinal (GI) B-cell lymphoma has been reported predominantly as a transmural lesion involving the stomach, jejunum, and ICCJ [4].

Clinically, evaluation of feline intestinal masses follows a stepwise approach incorporating diagnostic imaging, cytology, and histopathology. Abdominal ultrasonography is useful for lesion characterization, but it cannot establish tumor lineage on its own [2]. Focal mural masses associated with intermediate- to high-grade alimentary lymphoma may be reasonably diagnosed by ultrasound-guided fine-needle aspiration biopsy (FNAB) or mesenteric lymph node FNAB; however, limitations arise when cytologic atypia is unusual or when architectural information is required [2,3]. Moreover, the jejunum and ileum/ICCJ are important sites of feline intestinal lymphoma but are difficult to sample adequately by endoscopy, and lymphoid infiltration may extend beyond the mucosa into the submucosa, muscularis, and serosa; therefore, mucosal endoscopic biopsy alone may underestimate disease [5,6]. Accordingly, full-thickness histology and immunophenotypic evaluation are key steps in improving diagnostic accuracy, particularly when cytology or limited biopsy specimens are inconclusive [5,6].

In the present case, cytology and initial histopathology of an ICCJ mass suggested an intestinal NET, but immunohistochemistry (IHC) led to reclassification as B-cell lymphoma, most consistent with an intermediate-cell type. In humans, lymphoma has rarely been reported to exhibit NET-like morphology, creating a potential diagnostic pitfall on routine histopathologic evaluation [7]. However, to the authors’ knowledge, this is the first report of feline enteric, specifically ileocecocolic, B-cell lymphoma that mimicked an intestinal NET on both cytology and routine histopathology. This report illustrates the morphologic spectrum of feline intestinal B-cell lymphoma, highlights a clinically relevant diagnostic pitfall and describes multimodal management and prognostic considerations.

## 2. Case Description

A 3.3 kg, 8-year-old spayed female Korean Shorthair cat with a body condition score (BCS) of 3/9 was referred for evaluation of a history of at least 22 months of waxing and waning hyporexia, weight loss, and chronic vomiting. According to the referring hospital records, no diagnostic imaging had been performed, and chronic pancreatitis had been suspected and treated empirically 8 months earlier on the basis of vomiting and a feline pancreatic lipase (fPL) concentration near the upper end of the reference interval (RI) (3.2 µg/L).

**Figure 1:**
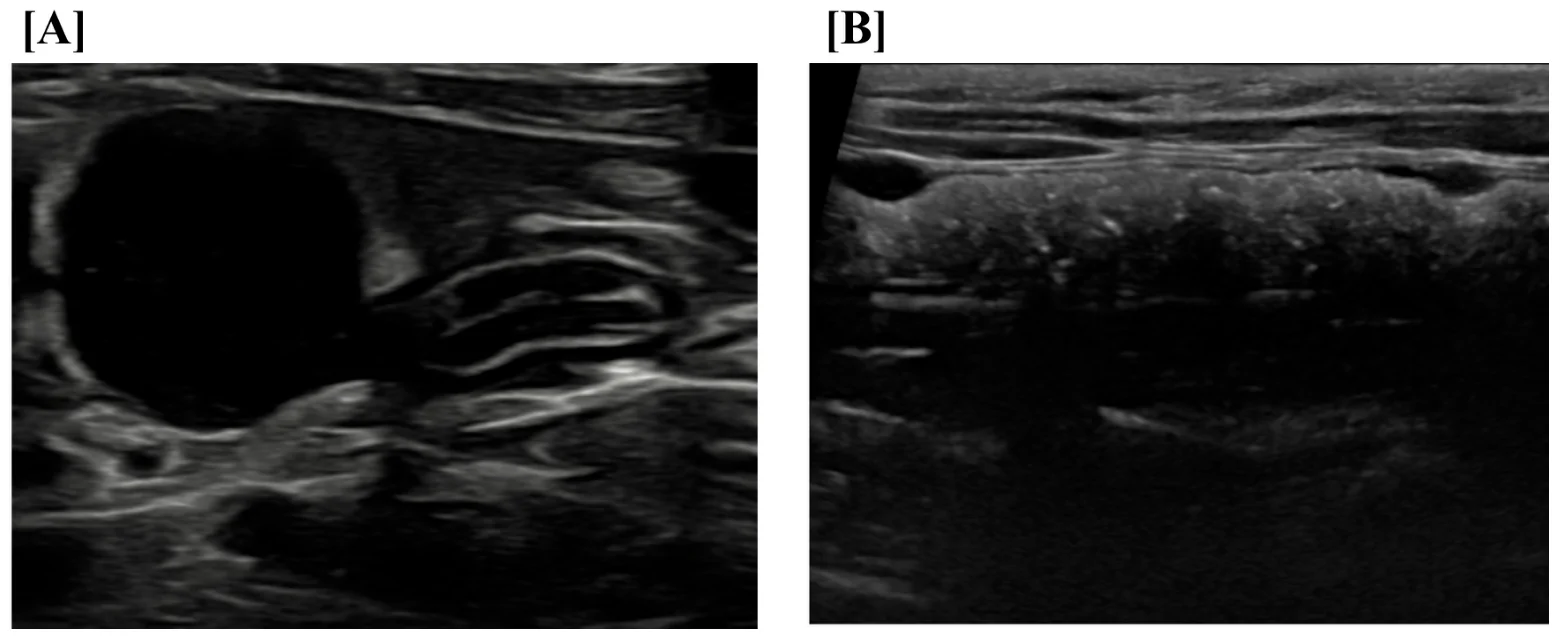
Preoperative ultrasonographic findings. (**A**) Focal mass at the ileocecocolic junction (ICCJ) contiguous with the bowel wall, (**B**) Multiple small nodular lesions associated with the colon.

At presentation, physical examination was unremarkable. Thoracic and abdominal radiographs were unremarkable, and FeLV/FIV testing was negative. A complete blood count (CBC) revealed neutrophilic leukocytosis (leukocytes, 26.82 × 10^3^/µL [RI, 5.5–19.5 × 10^3^/µL]; neutrophils, 23.15 × 10^3^/µL [RI, 2.5–12.5 × 10^3^/µL]), whereas hematocrit, hemoglobin concentration, and platelet count were within the reference intervals. Serum biochemistry revealed mild hyperglobulinemia (globulin, 5.3 g/dL [RI, 2.5–5.1 g/dL]) without other clinically relevant abnormalities, and electrolyte concentrations were within normal limits. The fPL concentration was 1.9 µg/L (RI, 0–3.5 µg/L), lower than the value recorded 8 months earlier (3.2 µg/L), and was within the RI. Abdominal ultrasonography revealed a 1.5 × 1.6 cm mass at the ICCJ that was continuous with the adjacent bowel wall (Figure 1A), together with multiple scattered colonic nodules measuring up to approximately 5 mm (Figure 1B).

**Figure 2:**
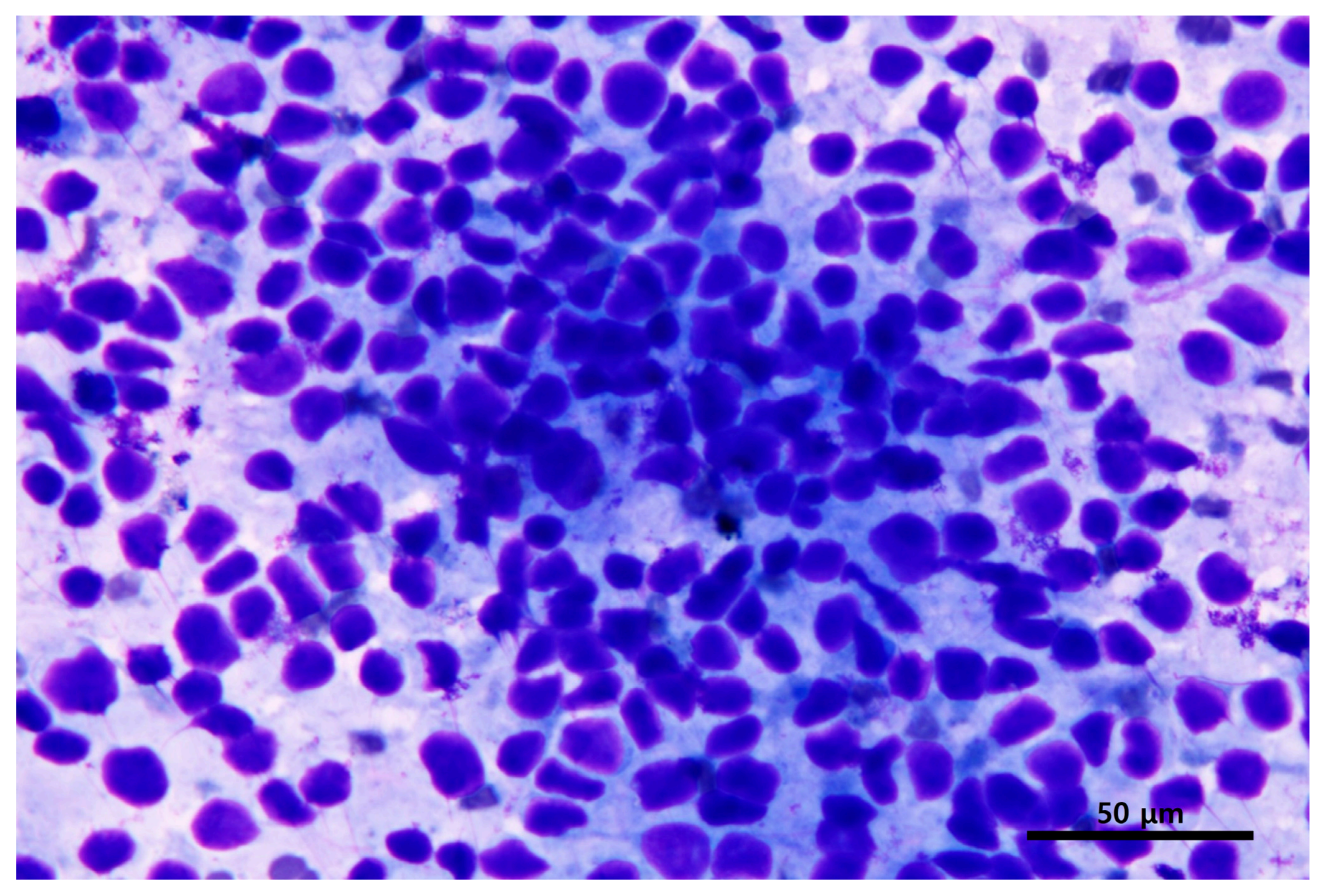
Cytologic findings of the ICCJ mass. Predominantly individually exfoliated oval cells with focal loosely cohesive cellular clustering (Diff Quick, original magnification × 400; scale bar = 50 μm).

FNAB of the ICCJ mass was performed. Cytologic evaluation revealed moderate-to-high cellularity composed predominantly of individually exfoliated oval cells, with loosely cohesive clusters in some areas. The cells had moderately basophilic cytoplasm and a moderate-to-moderately high nuclear-to-cytoplasmic (N:C) ratio. Moderate anisocytosis and anisokaryosis were also present (Figure 2). Based on these findings, a NET was considered the leading differential diagnosis, and histopathologic evaluation was recommended.

**Figure 3:**
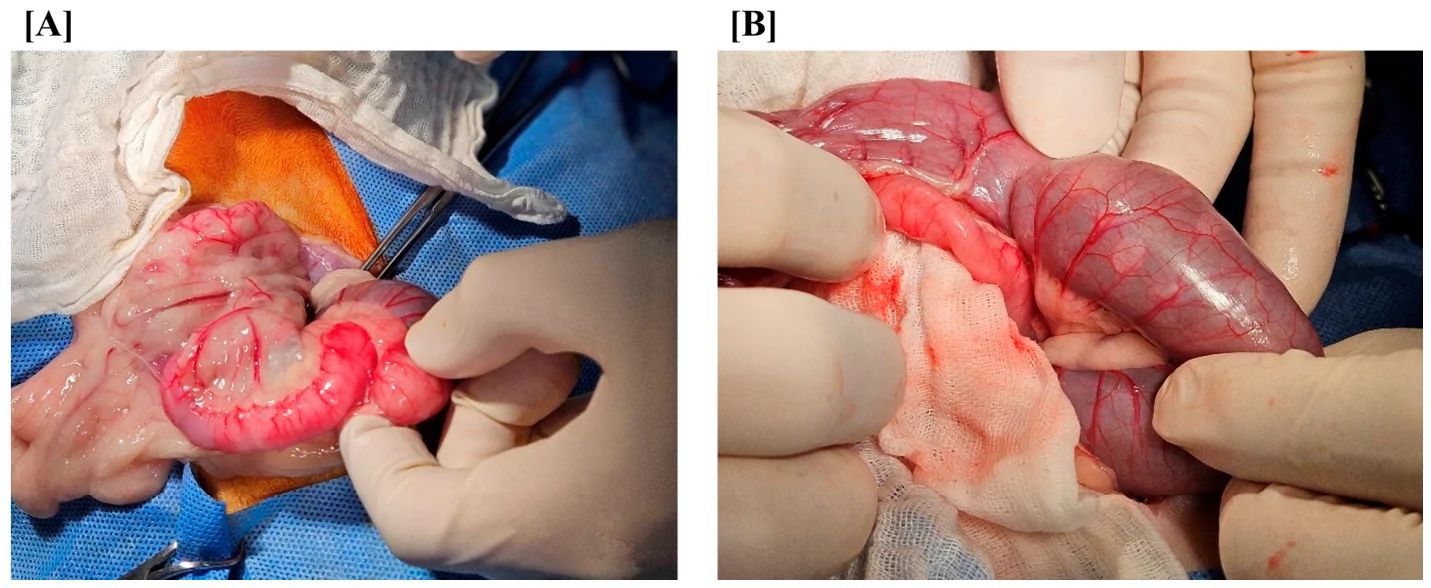
Intraoperative gross findings. (**A**) Focal mass at ICCJ, (**B**) Multiple small nodular lesions throughout the colon.

Supportive medical management, including an antiemetic (maropitant, 1 mg/kg, PO, q24h), an antibiotic (amoxicillin/clavulanate, 12.5 mg/kg, PO, q12h), and an acid-suppressive medication (famotidine, 0.5 mg/kg, PO, q12h), was administered for 7 days after presentation; however, intermittent vomiting persisted and no marked clinical improvement was observed. Preoperative computed tomography-based staging was considered but not performed because of owner-related constraints; therefore, surgical planning was based on ultrasonographic and cytologic findings. Eight days after presentation, based on the imaging and cytologic findings, the ICCJ mass was considered the dominant lesion amenable to resection, and exploratory laparotomy with segmental intestinal resection and anastomosis was performed. An intestinal segment >8 cm long, encompassing the distal ileum, ICCJ mass, and proximal colon was resected (Figure 3A). Multiple small nodular lesions were grossly visible throughout the colon (Figure 3B). At the time of surgery, the dominant ICCJ mass had been cytologically favored to represent a NET, and, in this diagnostic context, the colonic nodules were considered most consistent with possible reactive lymphoid follicles or lymphoid hyperplasia. Based on this interpretation, the colonic nodules were not histologically sampled. Because the nodules were numerous, multifocal, and widely distributed, complete excision within practical surgical margins was not considered feasible; therefore, surgical resection was directed toward the dominant ICCJ mass. Their pathologic nature remained undetermined.

Intestinal continuity was restored by side-to-side ileocolic anastomosis using a gastrointestinal anastomosis stapler (GIA; Covidien, Mansfield, MA, USA), and the intraoperative leak test was negative. The resected tissue was submitted for histopathologic evaluation. During hospitalization, no vomiting was observed. Mild diarrhea developed, but no other abnormal clinical signs or postoperative complications were noted. The cat was discharged on postoperative day 5.

**Figure 4:**
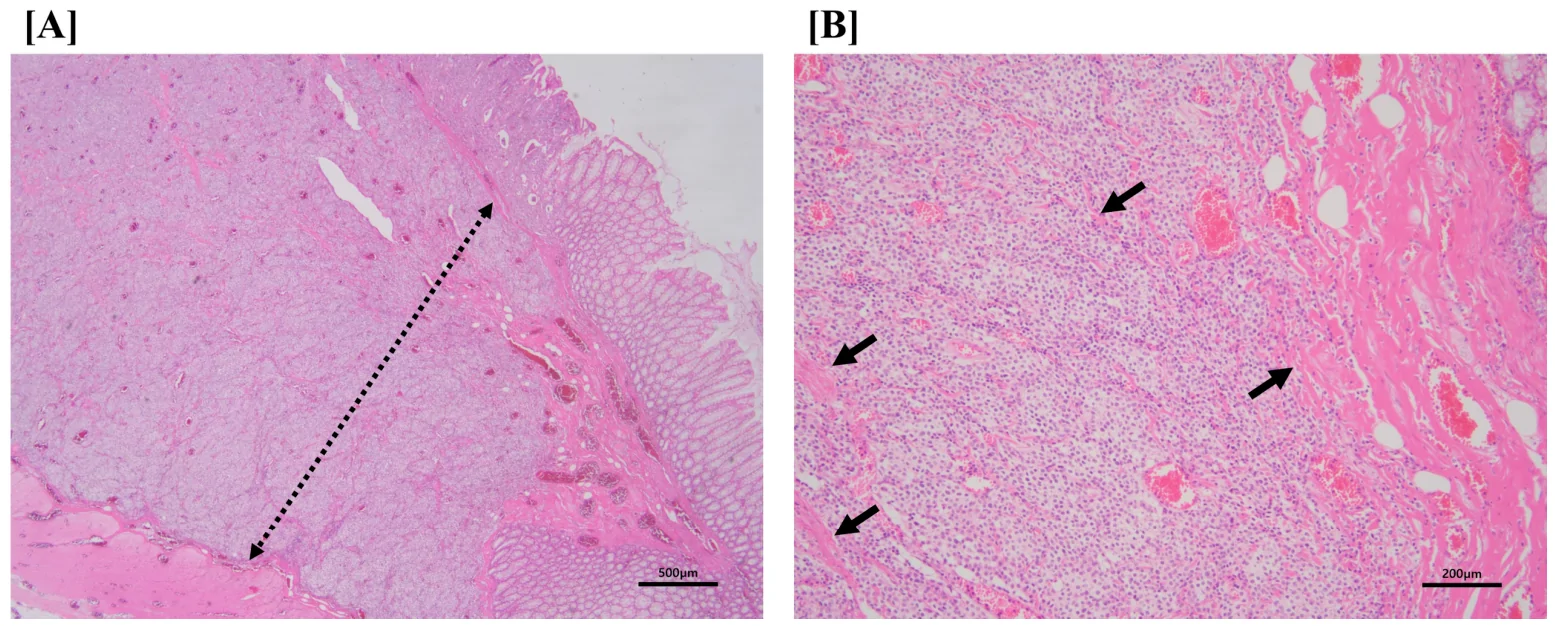
Histopathologic features of resected ICCJ mass. (**A**) A large submucosal mass (dashed line) beneath relatively preserved mucosa (hematoxylin and eosin [H&E], original magnification ×40; scale bar = 500 μm), (**B**) Broad submucosal clusters of neoplastic cells separated by fibrovascular stroma (arrows) (H&E, original magnification ×100; scale bar = 200 μm).

**Figure 5:**
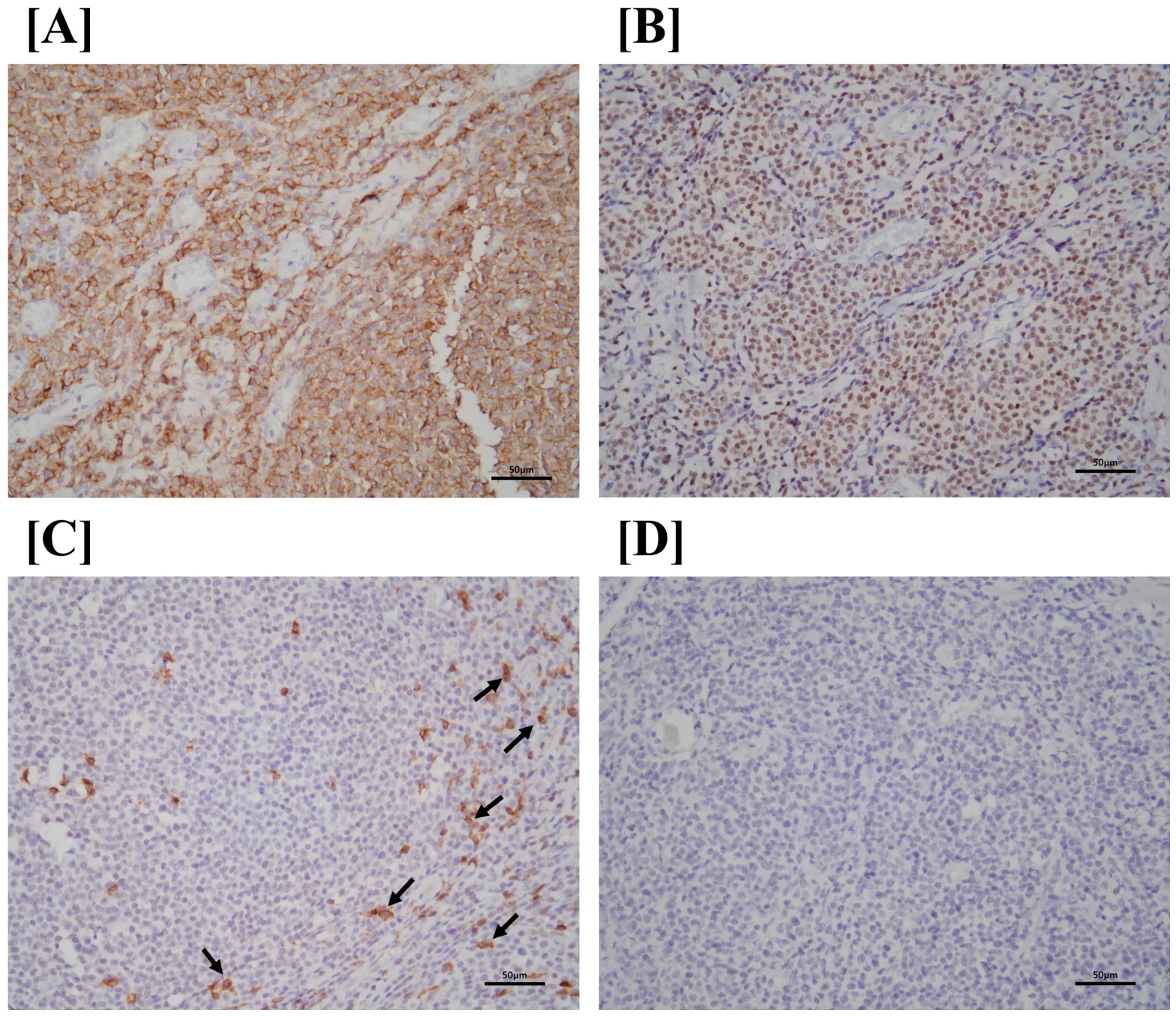
Immunohistochemical (IHC) characterization of ICCJ mass. (**A**) Strong CD20 immunoreactivity in neoplastic cells, (**B**) Strong nuclear PAX5 labeling in neoplastic cells, (**C**) Absence of CD3 immunoreactivity in neoplastic cells, with CD3-positive lymphocytes (arrows) located predominantly at the periphery of the neoplastic population, (**D**) Absence of chromogranin A immunoreactivity in neoplastic cells. Signals were visualized with DAB. Original magnification ×400; Scale bars = 50 μm.

Histopathologic examination of the resected specimen revealed a densely cellular proliferation of round-to-polygonal neoplastic cells. The neoplastic cells were relatively monomorphic and contained a moderate amount of eosinophilic to chromophobic granular cytoplasm. The nuclei were predominantly approximately two erythrocyte diameters in size, with occasional cells approaching 2–3 erythrocyte diameters, and contained 0–2 distinct nucleoli. The mitotic count was 12 per 10 high-power fields. The neoplastic cells formed large submucosal clusters reminiscent of prominent mucosa-associated lymphoid tissue (MALT), but the overall architecture was not considered typical of feline GI lymphoma (Figure 4). Although overt transmural proliferation was not identified, the lesion was widely distributed, and neoplastic infiltration was also present in the submucosa beyond the grossly evident mass. Neoplastic cells did not extend to the cut ends of the specimen; however, the minimum histologic margin was >0.52 mm. Mesenteric lymph nodes included in the submitted specimen were interpreted as reactively enlarged, and no definite metastatic infiltration by neoplastic cells was identified. Overall, the histomorphologic findings favored an intestinal NET. Therefore, IHC was performed using CD3 as a T-cell marker, CD20 and PAX5 as B-cell markers, and chromogranin A, synaptophysin, and NSE as neuroendocrine markers to distinguish the lesion from lymphoma and to assess neuroendocrine differentiation. The neoplastic cells showed strong CD20 immunoreactivity (Figure 5A) and strong nuclear immunoreactivity for PAX5 (Figure 5B). The neoplastic cells lacked CD3 immunoreactivity, whereas CD3-positive lymphocytes were located predominantly at the periphery of the neoplastic population (Figure 5C). In contrast, chromogranin A labeling was not detected in the neoplastic population (Figure 5D), and synaptophysin (Figure S1A) and NSE (Figure S1B) labeling were also absent. Taken together, these findings supported reclassification of the lesion as B-cell lymphoma with NET-like morphology, most consistent with an intermediate-cell type.

After discharge, vomiting was markedly reduced compared with the preoperative status and occurred only rarely despite discontinuation of antiemetic medication. On postoperative day 22, cyclophosphamide, doxorubicin, vincristine, and prednisolone (CHOP)-based chemotherapy was initiated. Following chemotherapy, no further vomiting was reported, even without antiemetic medication. At the most recent follow-up, approximately 6 months after surgery and completion of the third CHOP cycle, body weight had remained stable and no postoperative complications, including chronic diarrhea or clinical signs suggestive of anastomotic stricture, had been identified. Follow-up ultrasonography did not reveal a discrete mass suggestive of local recurrence at the surgical site. The multifocal colonic nodules were subjectively considered similar in size to those recorded at initial presentation.

## 3. Discussion

A noteworthy aspect of the present case is that the lesion did not conform to the cytologic pattern typically considered supportive of feline GI lymphoma at the FNAB stage. In cats with intestinal lymphoid disease, cytology may help identify a monomorphic lymphoid population or exclude other important differentials, but atypical lesions often cannot be confidently classified without histologic context and ancillary testing [2,8]. In the present case, the ICCJ aspirate was dominated by oval rather than round cells, showed only a moderate to moderately high N:C ratio, and, most notably, contained loosely cohesive cellular clusters in some fields. Collectively, these features produced a cytologic picture that was not overtly suggestive of a conventional lymphoid neoplasm and explained why a NET was initially favored. Feline GI lymphoma is often dominated by small-intestinal T-cell disease, while GI B-cell lymphomas represent a less common subset that may manifest as mass-forming, transmural lesions involving the stomach, jejunum, or ICCJ [4]. The cytologic profile observed in the present case therefore lay outside the range usually invoked to support a lymphoid diagnosis, suggesting that the unusual preoperative differential reflected the tumor’s atypical cytomorphology rather than a simple interpretive error.

The histologic appearance of the present lesion reinforced, rather than resolved, this diagnostic ambiguity. Feline GI B-cell lymphomas are most commonly diffuse large B-cell lymphomas with transmural infiltration, whereas the present tumor was characterized by an intermediate-cell phenotype forming MALT-like submucosal clusters in the absence of the diffuse large-cell pattern typical of this entity [4]. This discordance is particularly relevant from a veterinary pathology standpoint, because direct translation of the human World Health Organization (WHO) GI lymphoma nomenclature to cats has been shown to be imperfect, with feline cases frequently failing to map cleanly onto human diagnostic categories [9]. Accordingly, based on the histologic and immunophenotypic findings, the lesion is best regarded as an unusual intermediate-cell B-cell lymphoma with NET-like morphology rather than as a straightforward feline analogue of the WHO classification [9]. Recent work has further emphasized immunophenotypic heterogeneity among feline intestinal lymphomas, particularly in non-B-cell mass-forming cases, supporting immunophenotypic confirmation when morphology is unusual [10]. Taken together, although B-cell lymphoma at the ICCJ is anatomically plausible, the NET-like impression in this case appeared to result from a combination of several atypical morphologic features. Cytologically, the predominance of oval rather than round cells and the presence of focal loosely cohesive clusters, rather than discrete non-cohesive cells, made the lesion less suggestive of a lymphoid neoplasm. Histologically, relative cellular monomorphism, eosinophilic-to-chromophobic granular cytoplasm, broad submucosal clustering, and fibrovascular separation created an architectural appearance resembling a NET. In a reported human mediastinal large B-cell lymphoma with rosette formation mimicking thymic carcinoid, the rosette centers were composed of interdigitating cytoplasmic processes, and cytoskeletal redistribution and altered cell–cell or cell–matrix interactions were proposed as possible mechanisms [7]. However, because definite rosette formation was not observed, whether similar mechanisms contributed to the NET-like morphology in the present case remains unknown.

In light of these atypical cytologic and histologic findings, the central diagnostic issue in this case was lineage assignment and assessment for neuroendocrine differentiation. An intestinal mast cell tumor with atypical morphology could also be considered as an additional morphologic differential diagnosis, because feline intestinal mast cell tumors may show atypical morphology that complicates their distinction from lymphoma and neuroendocrine carcinoma [11]. However, IHC demonstrated CD20 immunoreactivity and strong nuclear PAX5 immunoreactivity in the neoplastic cells. The neoplastic cells lacked CD3 immunoreactivity, whereas CD3-positive lymphocytes were located predominantly at the periphery of the neoplastic population. Labeling for chromogranin A, synaptophysin, and NSE was absent. The combined PAX5 and CD20 immunoreactivity supported B-cell lineage, as CD20 is used as a B-cell marker in feline intestinal lymphoma and PAX5 has been shown to label feline B-cell lymphomas but not feline T-cell lymphomas [10,12]. The absence of chromogranin A, synaptophysin, and NSE labeling provided no immunohistochemical evidence of neuroendocrine differentiation, and interpretation was based on the overall panel rather than on a single neuroendocrine marker alone [13]. On the basis of this panel-based interpretation, the diagnosis was revised from the initially favored intestinal NET to B-cell lymphoma with NET-like morphology. This reclassification could not have been possible by morphologic assessment alone. Accordingly, in feline intestinal lesions whose cytologic or histologic features are insufficient for confident lineage assignment, confirmatory lineage assessment by IHC—and, for cytologic specimens, ICC or cell-block-based immunophenotyping—should be strongly considered as adjuncts when adequate material is available [14].

Even after reclassification as lymphoma, the surgical approach in this case may still be regarded as clinically justified. In general, systemic chemotherapy remains the mainstay of treatment for feline intermediate- or large-cell GI lymphoma, and lymphoma is not typically approached as a purely local neoplasm amenable to wide oncologic excision. However, when a lesion presents as a discrete mass-forming process and preoperative cytology favors a non-lymphoid intestinal tumor over lymphoma, as in the present case, segmental resection may have diagnostic value and a potential role in local disease management. At the time of surgical decision-making, the dominant ICCJ mass had been cytologically favored to represent a NET. In that preoperative diagnostic context, the multifocal colonic nodules were considered more compatible with reactive lymphoid follicles or lymphoid hyperplasia than with definitive evidence of diffuse neoplastic disease. Similar micronodular lesions have been described ultrasonographically in the colonic submucosa and proposed to represent reactive intraparietal lymphoid follicles [15]. However, because these colonic nodules were not histologically sampled, their pathologic nature remains uncertain. They may have represented additional lymphoma foci, reactive or inflammatory lymphoid nodules, or another process unrelated to the primary ICCJ mass. Therefore, the extent of disease and the contribution of surgery to local disease control should be interpreted cautiously. In such circumstances, surgery can provide diagnostic tissue and facilitate local management of the dominant lesion, and may mitigate local mass-related concerns such as obstruction or perforation before chemotherapy [16,17,18]. Retrospective studies of resection followed by CHOP and of surgical resection of discrete GI lymphoma masses support the clinical plausibility of surgery-containing multimodal management in selected cases [17,18,19], whereas post-chemotherapy GI perforation has been reported in cats managed medically for discrete intermediate- or large-cell GI lymphoma [16]. Although this clinical course does not establish that surgery prevented subsequent complications, the marked reduction in vomiting before chemotherapy suggests that resection may have contributed to short-term clinical improvement. However, because this was a single case, CHOP-based chemotherapy was subsequently administered, and the pathologic nature of the multifocal colonic nodules remained undetermined, the independent contribution of surgery to long-term local disease control or survival cannot be determined. Accordingly, the primary roles of surgery in this case were to obtain full-thickness tissue for definitive diagnosis and to provide local disease management of the dominant ICCJ lesion before systemic therapy. The postoperative course should not be interpreted as demonstrating a survival benefit attributable to surgery. In retrospect, the numerous small colonic lesions observed throughout the colon at surgery also caution against interpreting the disease as a strictly local, margin-centered process once lymphoma was confirmed; however, because these lesions were not biopsied, their relationship to the confirmed lymphoma remains presumptive. Therefore, the close histologic margin (>0.52 mm) should be interpreted not simply as under-resection, but in the context of lymphoma biology, in which microscopic extension or multifocal involvement may extend beyond the grossly dominant ICCJ mass [20].

Because resection included the ICCJ, postoperative gastrointestinal function was monitored, as loss of this junction may be associated with altered transit and other gastrointestinal consequences [21,22,23]. Intestinal continuity was restored using a stapled side-to-side enterocolic anastomosis, a technique previously evaluated in cats [24]. Although the outcome of a single case cannot establish the superiority of this reconstructive technique, no clinically important postoperative diarrhea, progressive weight loss, or clinical evidence of anastomotic stricture was documented during the available follow-up period, indicating a clinically favorable functional outcome in this cat.

This case report has several limitations. First, preoperative computed tomography-based staging was not performed because of owner-related constraints; therefore, the full extent of abdominal disease could not be assessed before surgery. Second, because the multifocal colonic nodules observed intraoperatively and ultrasonographically were not histologically sampled, their relationship to the confirmed ICCJ lymphoma and the overall extent of disease remain uncertain. Third, the follow-up period was limited to approximately 6 months, which is insufficient to evaluate long-term disease control, recurrence, or survival. Finally, as this is a single case report, the diagnostic and therapeutic implications should be interpreted cautiously and cannot be generalized without further case accumulation.

## 4. Conclusions

This case demonstrates that feline intestinal B-cell lymphoma may rarely exhibit NET-like morphology, leading to misleading cytologic and routine histopathologic impressions. For intestinal masses that are not overtly lymphoid in appearance, particularly lesions at the ICCJ, where B-cell lymphoma is anatomically plausible but may be morphologically atypical, full-thickness tissue acquisition and IHC-based lineage confirmation should be incorporated, when feasible, into the diagnostic approach rather than relying on morphology alone [5,6]. Ultimately, this case is best understood not simply as B-cell lymphoma arising at the ICCJ, but as a demonstration that an enteric tumor that is not morphologically typical of lymphoma may nevertheless prove to be B-cell lymphoma upon definitive immunophenotypic evaluation.

## Data Availability

The data presented in this study are available on request from the corresponding author due to privacy, confidentiality, and ethical considerations related to the clinical records and images of a client-owned animal.

## Supplementary Materials

The following supporting information can be downloaded at: https://www.mdpi.com/article/10.3390/vetsci13080731/s1, Figure S1: Additional neuroendocrine immunohistochemical findings in the ICCJ mass.

## Abbreviations

The following abbreviations are used in this manuscript:

BCS	Body condition score
CBC	Complete blood count
CHOP	Cyclophosphamide, doxorubicin, vincristine, and prednisolone
FeLV	Feline leukemia virus
FIV	Feline immunodeficiency virus
FNAB	Fine-needle aspiration biopsy
fPL	Feline pancreatic lipase
GI	Gastrointestinal
GIA	Gastrointestinal anastomosis stapler
H&E	Hematoxylin and Eosin
ICCJ	Ileocecocolic junction
IHC	Immunohistochemistry
MALT	Mucosa-associated lymphoid tissue
N:C ratio	Nuclear-to-cytoplasmic ratio
NET	Neuroendocrine tumor
PO	Per os
RI	Reference interval
WHO	World Health Organization

## References

[1] Louwerens M., London C.A., Pedersen N.C., Lyons L.A. (2005). Feline lymphoma in the post-feline leukemia virus era. J. Vet. Intern. Med..

[2] Barrs V.R., Beatty J.A. (2012). Feline alimentary lymphoma: 1. Classification, risk factors, clinical signs and non-invasive diagnostics. J. Feline Med. Surg..

[3] Barrs V.R., Beatty J.A. (2012). Feline alimentary lymphoma: 2. Further diagnostics, therapy and prognosis. J. Feline Med. Surg..

[4] Moore P.F., Rodriguez-Bertos A., Kass P.H. (2012). Feline gastrointestinal lymphoma: Mucosal architecture, immunophenotype, and molecular clonality. Vet. Pathol..

[5] Kiupel M., Smedley R.C., Pfent C., Xie Y., Xue Y., Wise A.G., DeVaul J.M., Maes R.K. (2011). Diagnostic algorithm to differentiate lymphoma from inflammation in feline small intestinal biopsy samples. Vet. Pathol..

[6] Evans S.E., Bonczynski J.J., Broussard J.D., Han E., Baer K.E. (2006). Comparison of endoscopic and full-thickness biopsy specimens for diagnosis of inflammatory bowel disease and alimentary tract lymphoma in cats. J. Am. Vet. Med. Assoc..

[7] Tsai H.W., Yen Y.S., Chang K.C. (2006). Mediastinal large B-cell lymphoma with rosette formation mimicking thymoma and thymic carcinoid. Histopathology.

[8] Marsilio S., Freiche V., Johnson E., Leo C., Langerak A.W., Peters I., Ackermann M.R. (2023). ACVIM consensus statement guidelines on diagnosing and distinguishing low-grade neoplastic from inflammatory lymphocytic chronic enteropathies in cats. J. Vet. Intern. Med..

[9] Wolfesberger B., Fuchs-Baumgartinger A., Greß V., Hammer S., Gradner G., Knödl K., Tichy A., Rütgen B., Beham-Schmid C. (2018). World Health Organisation Classification of Lymphoid Tumours in Veterinary and Human Medicine: A Comparative Evaluation of Gastrointestinal Lymphomas in 61 Cats. J. Comp. Pathol..

[10] Wolfesberger B., Gradner G., Rütgen B.C., Hittmair K.M., Walter I., Donovan T.A., Kleiter M., Krischak A., Burgener I.A., Fuchs-Baumgartinger A. (2024). Immunophenotype investigation in feline intestinal non-B-cell lymphoma. J. Comp. Pathol..

[11] Sabattini S., Giantin M., Barbanera A., Shahidian L.Z., Dacasto M., Zancanella V., Prata D., Trivigno E., Bettini G. (2016). Feline intestinal mast cell tumours: Clinicopathological characterisation and KIT mutation analysis. J. Feline Med. Surg..

[12] Felisberto R., Matos J., Alves M., Cabeçadas J., Henriques J. (2017). Evaluation of Pax5 expression and comparison with BLA.36 and CD79alphacy in feline non-Hodgkin lymphoma. Vet. Comp. Oncol..

[13] Ferreira-Neves P., Lezmi S., Lejeune T., Rakotovao F., Dally C., Fontaine J.-J., Bernex F., Cordonnier N. (2008). Immunohistochemical characterization of a hepatic neuroendocrine carcinoma in a cat. J. Vet. Diagn. Investig..

[14] Sampaio F., Marrinhas C., Oliveira L.F., Malhão F., Lopes C., Gregório H., Correia-Gomes C., Marcos R., Caniatti M., Santos M. (2023). Detection of Lymphoid Markers (CD3 and PAX5) for Immunophenotyping in Dogs and Cats: Comparison of Stained Cytology Slides and Matched Cell Blocks. Vet. Sci..

[15] Citi S., Chimenti T., Marchetti V., Millanta F., Mannucci T. (2013). Micronodular ultrasound lesions in the colonic submucosa of 42 dogs and 14 cats. Vet. Radiol. Ultrasound.

[16] Crouse Z., Phillips B., Flory A., Mahoney J., Richter K., Kidd L. (2018). Post-chemotherapy perforation in cats with discrete intermediate- or large-cell gastrointestinal lymphoma. J. Feline Med. Surg..

[17] Tidd K.S., Durham A.C., Brown D.C., Velovolu S., Nagel J., Krick E.L. (2019). Outcomes in 40 cats with discrete intermediate- or large-cell gastrointestinal lymphoma masses treated with surgical mass resection (2005–2015). Vet. Surg..

[18] Gouldin E.D., Mullin C., Morges M., Mehler S.J., de Lorimier L., Oakley C., Risbon R., May L., Kahn S.A., Clifford C. (2017). Feline discrete high-grade gastrointestinal lymphoma treated with surgical resection and adjuvant CHOP-based chemotherapy: Retrospective study of 20 cases. Vet. Comp. Oncol..

[19] Holenova K., Odatzoglou P., Taylor F. (2025). A retrospective descriptive study of colorectal large or intermediate cell lymphoma in cats managed with surgical resection and/or medical management. J. Feline Med. Surg..

[20] Morrice M., Polton G., Beck S. (2019). Evaluation of the histopathological extent of neoplastic infiltration in intestinal tumours in cats. Vet. Med. Sci..

[21] Sweet D.C., Hardie E.M., Stone E.A. (1994). Preservation versus excision of the ileocolic junction during colectomy for megacolon: A study of 22 cats. J. Small Anim. Pract..

[22] Fernandez Y., Seth M., Murgia D., Puig J. (2017). Ileocolic junction resection in dogs and cats: 18 cases. Vet. Q..

[23] Stecyk C.N., Freeman L.M., Webster C.R.L., Penninck D.G., Marino K., Berg J. (2022). Gastrointestinal signs and a need for nutritional management may persist long term in dogs and cats undergoing resection of the ileocolic junction: 35 cases (2008–2020). J. Am. Vet. Med. Assoc..

[24] Costello S., McRae B., Olive M., A Marks T., Mielke B., Billet J.-P., Levien A., Basa R.M. (2024). Stapled enterectomy reduces surgical time when compared with sutured enterectomy: A retrospective review of 54 cats. J. Feline Med. Surg..

