# Supplementary material for: Ileocecocolic B-Cell Lymphoma Mimicking an Intestinal Neuroendocrine Tumor in a Cat: A Diagnostic Pitfall
Source: Vet Sci. 2026 Jul 24;13(8):731. doi: 10.3390/vetsci13080731 (PMC13517762; doi:10.3390/vetsci13080731)
Supplement: Supplementary file 1 [file vetsci-13-00731-s001.zip › vetsci-4427538-supplementary.pdf]

Case Report

Ileocecocolic B-Cell Lymphoma Mimicking an Intestinal Neuroendocrine Tumor in a Cat: A Diagnostic Pitfall

Ha-Neul Cho , Hyo-Sung Kim , Ki-Jung Kim and Hwi-Yool Kim *

[A] **[B]**

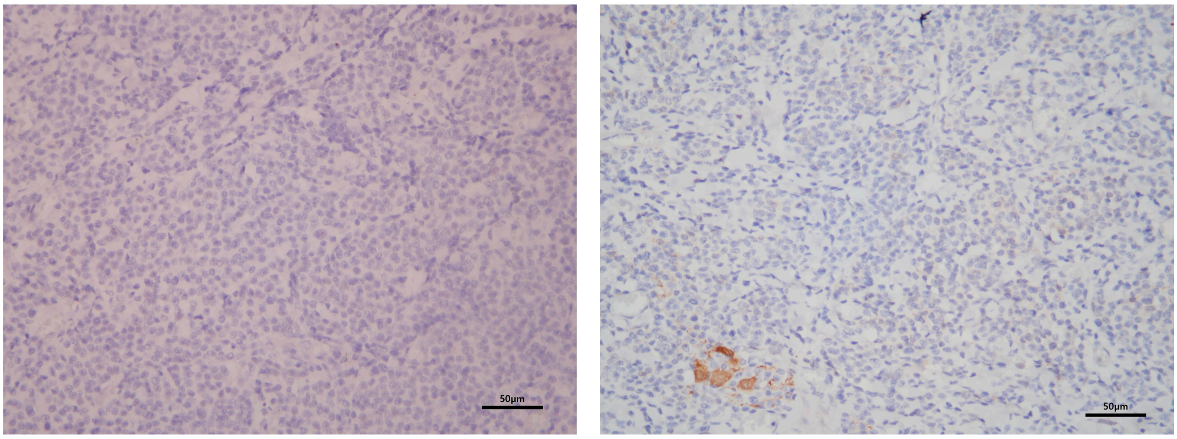

Figure S1: Additional neuroendocrine immunohistochemical findings in the ICCJ mass.
